# Development of a Finite Element Model of Decompressive Craniectomy

**DOI:** 10.1371/journal.pone.0102131

**Published:** 2014-07-15

**Authors:** Tim L. Fletcher, Angelos G. Kolias, Peter J. A. Hutchinson, Michael P. F. Sutcliffe

**Affiliations:** 1 Department of Engineering, University of Cambridge, Cambridge, United Kingdom; 2 Division of Neurosurgery, Department of Clinical Neurosciences, Addenbrooke's Hospital & University of Cambridge, Cambridge, United Kingdom; Glasgow University, United Kingdom

## Abstract

Decompressive craniectomy (DC), an operation whereby part of the skull is removed, is used in the management of patients with brain swelling. While the aim of DC is to reduce intracranial pressure, there is the risk that brain deformation and mechanical strain associated with the operation could damage the brain tissue. The nature and extent of the resulting strain regime is poorly understood at present. Finite element (FE) models of DC can provide insight into this applied strain and hence assist in deciding on the best surgical procedures. However there is uncertainty about how well these models match experimental data, which are difficult to obtain clinically. Hence there is a need to validate any modelling approach outside the clinical setting. This paper develops an axisymmetric FE model of an idealised DC to assess the key features of such an FE model which are needed for an accurate simulation of DC. The FE models are compared with an experimental model using gelatin hydrogel, which has similar poro-viscoelastic material property characteristics to brain tissue. Strain on a central plane of the FE model and the front face of the experimental model, deformation and load relaxation curves are compared between experiment and FE. Results show good agreement between the FE and experimental models, providing confidence in applying the proposed FE modelling approach to DC. Such a model should use material properties appropriate for brain tissue and include a more realistic whole head geometry.

## Introduction

Uncontrolled brain swelling and raised intra-cranial pressure can lead to death or poor functional outcome in patients suffering from severe traumatic brain injury, ischaemic stroke and other type of brain insults [1], [2]. Decompressive craniectomy (DC) is a surgical procedure which involves the following steps: opening of the skin (scalp incision), removal of a large piece of skull (bone flap), opening of the outermost membrane covering the brain (dura mater) and closure of the skin incision. The creation of an opening in the skull essentially turns the cranial cavity from non-compliant into compliant [3]. As a result, intra-cranial pressure is reduced after a DC [4], [5]. A number of clinical studies have been launched recently in an attempt to collate high-quality evidence regarding the clinical effectiveness of this operation in patients suffering from traumatic brain injury and ischaemic stroke [6]–[8]. While evidence is still accumulating, it is important that further work is undertaken in an attempt to better characterise the effects of DC on the brain. This is especially important for clinicians, as the optimal parameters of craniectomy (particularly size and location) remain controversial [9].

In the context of brain injury and swelling, DC will induce mechanical strain in the brain tissue which may cause damage [10]. Engineering models of DC can provide predictions of the induced strain and hence identify regions of the brain in which damage may occur. Although there have been various finite element (FE) models of the brain during surgery (e.g.[11]), there is only one model in the literature that focuses on decompressive craniectomy [12]. Gao and Ang [12] present a three dimensional model of the brain and skull with material properties taken from Cheng and Bilston [13]. However their model does not consider how details of the material model or geometric features affect the accuracy of the predicted strain fields in the brain, nor do they provide experimental validation. Indeed clinical data to support such models are difficult to obtain, reinforcing the need for a careful validation study of the FE approach before applying it to simulate DC. Strains extracted from patient CT scans (see [14] for methods) could be used to compare full skull geometry models. However post-op CT scans generally take place a number of days after surgery; therefore early tissue response to the craniectomy would be lost.

One aspect that needs to be considered carefully is the type of material model required. In decompressive craniectomy there is a hybrid of confined and unconfined loading, which means that conclusions drawn on appropriate material models from other brain deformation studies may not be valid for DC. Kyriacou *et al.* [15] suggest that a poro-viscoelastic (PVE) model of the brain combines the advantages of both poroelastic models at low strain rates and viscoelastic models for high strain rates. PVE parameters for the brain are available from Cheng and Bilston [13] and Franceschini [16].

This paper aims to identify an appropriate FE model for DC. Experimental tests on an idealised DC geometry, using gelatin hydrogel to represent brain tissue, are compared with corresponding FE models with a PVE material model. Rather than adopting the complex geometry of the surgical situation, the approach used in this paper is to consider a simplified axisymmetric model of DC. Validation of the model is based on load response and strain comparisons between FE and experimental models. Good correlation between these experiments and FE models can provide confidence in adopting the proposed FE model, using material models representative of brain tissue and realistic head geometries, to analyse the effects of DC on the brain.

## Methods

Typically a DC has an approximately circular or elliptical (for unilaterial DC) geometry [9]. This suggest an idealised model of DC using a cylindrical geometry for the ‘skull’ and a circular craniectomy opening. The experimental model and FE approach are described below and results for the two approaches are then compared.

### Experimental model

**Figure 1:**
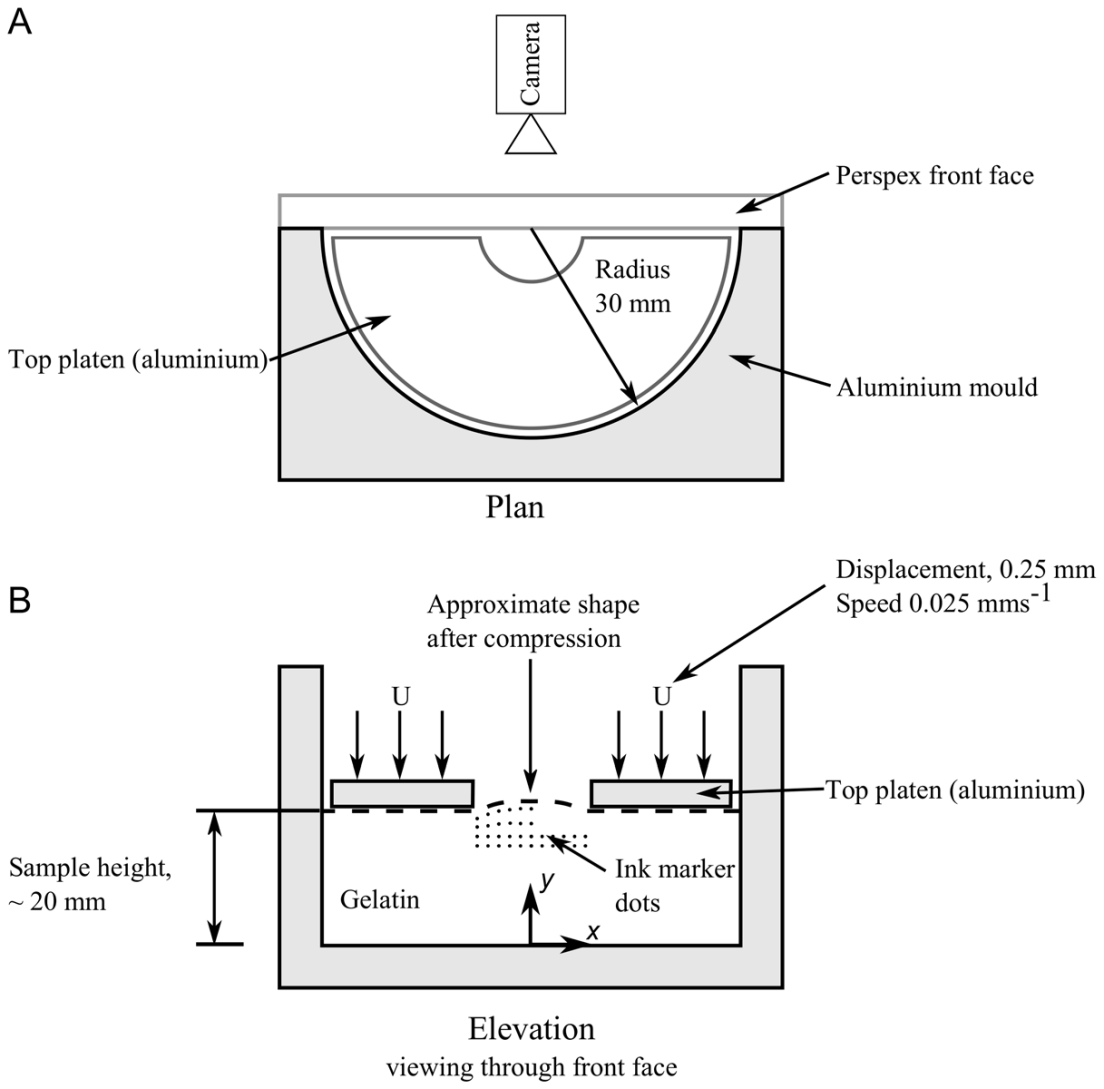
Schematic diagram of the experimental semi-cylindrical model. (A) plan, (B) elevation.

The experimental model of the idealised craniectomy with semi-cylindrical geometry of radius 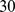 mm and height 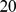 mm is illustrated in Figure 1. Gelatin was used to simulate the brain tissue, while a semi-circular aluminium mould modelled the constraint of the skull. A flat perspex front plate was in contact with the gelatin to constrain the movement while keeping the front face visible. It is known that gelatin hydrogels exhibit PVE behaviour [17] and gelatin has also been used as a surrogate for brain in experimental brain models [18], [19]. While not an exact match for brain tissue, gelatin provides insight into how a PVE material would respond under similar loading situations to DC. In clinical practice intra-cranial pressure causes deformation of the brain out through the craniectomy opening. In the experimental simulation this deformation is instead associated with a reduction in the size of the cavity, induced by movement of a top platen containing the semicircular craniectomy opening. There was a fillet radius 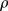 of 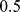 mm around the edge of the craniectomy opening and craniectomy radii of 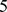, 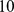 and 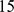 mm were used.

#### Manufacture

The experimental model used 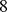
% 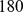 g bloom porcine gelatin hydrogels (Sigma Aldrich), made in a similar manner to the method described by Galli and Oyen [20]. Samples were created by casting molten gelatin into silicone moulds, acetate was then placed on the top surface preventing a meniscus forming. The gels were refrigerated at 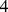
°C overnight for ease of extraction from the moulds.

After extraction, the gels were marked with dots of Indian Ink (Winsor and Newton) on the front face, see Figure 1. The application of these markers on the flat face allowed image analysis of the strain on a plane in the centre of the simulated craniectomy for comparison with the FE model.

The gels were left overnight in a sealed container for the ink to dry whilst minimising sample dehydration. The samples were tested 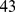 hours 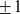 hour after the gels were originally cast.

### Materials characterisation

Gelatin hydrogels can be produced with varying concentrations of gelatin and water producing large variations in material parameters. Therefore it is necessary to characterise the specific gelatin hydrogels used. For this type of gelatin, confined compression tests approximately isolate the poro-elastic behaviour of the gelatin whilst unconfined compression tests approximately isolate the visco-elastic response.

Gels were manufactured in the same manner as for experimental tests. Cylindrical confined and unconfined compression samples were cast with radii of 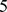 mm and heights of 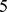 mm and 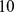 mm respectively. Unconfined compression tests were carried out in displacement control. Strain was applied over a 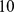 s ramp to a peak strain of 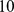
%, followed by a hold time of 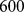 s. Confined compression tests were load controlled. After initial contact, load was increased over a 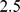 s ramp to a peak load of 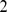 N which was held for 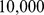 s.

The PVE material model used in this paper is a well-established approach to considering time-dependent material behaviour. The following paragraphs in this section provide an overview of the model and detail the procedures used to identify the material parameters. References are given for a more detailed description of the model.

The governing equations for a PVE material are as follows: the poro-elastic component of the response is characterised by the instantaneous Young's modulus 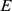 of the solid, the Poisson's ratio 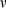 and a permeability 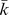. The time domain visco-elasticity of the solid is introduced by a relaxation function which is applied to the solid component of the material, as follows [21]: 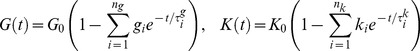
(1)


The 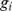 and 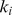 terms represent dimensionless shear and bulk relaxation moduli respectively and 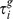 are corresponding time constants, with 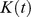 and 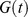 being time-dependent bulk and shear moduli. The PVE model used in this paper assumes 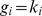 and 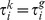 for all time constants. This is the same model as the “BPVE2” model used by Suh and DiSilvestro [22]. It is also assumed that the relative relaxation rates of visco-elasticity and poro-elasticity differ significantly in unconfined compression, so that the visco-elastic response can be determined from unconfined compression tests.

The unconfined compression tests were analysed using a standard three parameter Prony series form of the relaxation function to solve the Boltzmann hereditary integral [23], as per [24] by Equation 2. 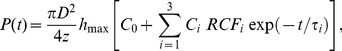
(2)where 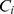 and 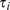 are the Prony parameters, 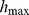 the maximum displacement, 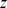 the sample height, 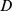 the sample diameter, 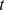 the time and 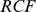 is a ramp correction factor defined as:



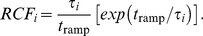
(3)Matlab (The Mathworks, Natick, MA, US) was used to identify the parameters 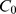, 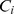 and 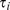 which best fit the measured responses using Equation 2. The dimensionless shear relaxation moduli 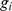 can be determined from the 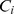 terms directly as 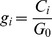.

Confined compression tests were analysed by curve-fitting the consolidation curves assuming a Terzaghi solution in a similar manner to Franceschini [16]. The Terzaghi solution takes the form: 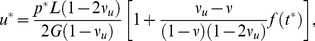
(4)where 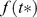 is:
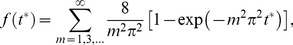
(5)following Detournay and Cheng [25]. Here the consolidation parameter 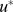 is a non-dimensional settling of the sample under constant load, with 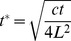 representing a non-dimensional time, 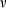 is the Poisson's ratio with 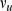 the undrained Poisson's ratio, 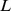 is the layer thickness and 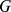 is the shear modulus. The constant 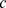 (assuming incompressible constituents) is given by:



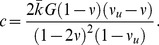
(6)Again Matlab was used to identify the material parameters 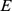, 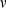 and 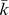 which best fit the measured response in the above equation.

Table 1 details the values of the poro-elastic and visco-elastic material parameters derived from these confined and unconfined compression tests.

**Table 1:** PVE gelatin material properties, derived from characterisation tests.

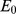 /kPa	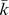 /mm s^−1^	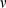	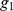	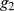	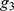	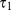 /s	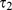 /s	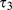 /s
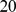	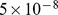	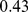	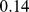	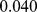	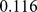	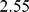	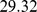	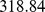

#### Test protocol

This section details the protocol used for the deformation of the idealised DC model illustrated in Figure 1. The aluminium platen was fixed to an Instron 5544 universal testing machine (Instron, UK) fitted with a 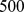 N load cell. A compressive pre-load of 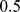 N was applied to the platen in order to locate the surface of the sample and fully confine the sample in the rig. The need for a preload was highlighted by Cheng and Bilston [13] (for unconfined compression of a PVE material). A total displacement of 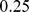 mm was then applied at a speed of 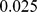 mm s^−1^, which is a similar speed to that used by Miller and Chinzei [26] and representative of surgical strain rates. This was followed by a hold period of 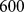 s at the final displacement of 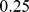 mm. This hold period provides substantial visco-elastic and poro-elastic relaxation of the gelatin without dehydrating the sample. All tests were carried out for a minimum of 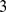 repeats per opening size.

#### Imaging

Images of the front face of the gelatin were captured at 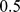 s intervals during testing using a camera (Pixelink, Ottawa, Canada) and analysed using Matlab.

The markers on the front face of the gelatin were tracked throughout the experiment using methods developed by Crocker [27]. The centres of the markers in each frame determine the path of the markers in successive images given constraints on the maximum distance each particle can travel.

Markers which were tracked for less than 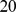
% of the frames were discarded as noise. The remaining paths were fitted with a second-order polynomial in both the 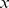 and 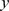 directions. This both smooths the output and interpolates the location of the markers in any frame where they are missing.

The resultant fitted paths of the markers were used to determine the applied strain on the front face. First, Delaunay triangulation [28] was used to define a triangular mesh which described the marker positions in the first time-step. This mesh was then used to find the strain in each element from the displacement of each marker using the methods of Screen and Evans [29].

### Finite element model

**Figure 2:**
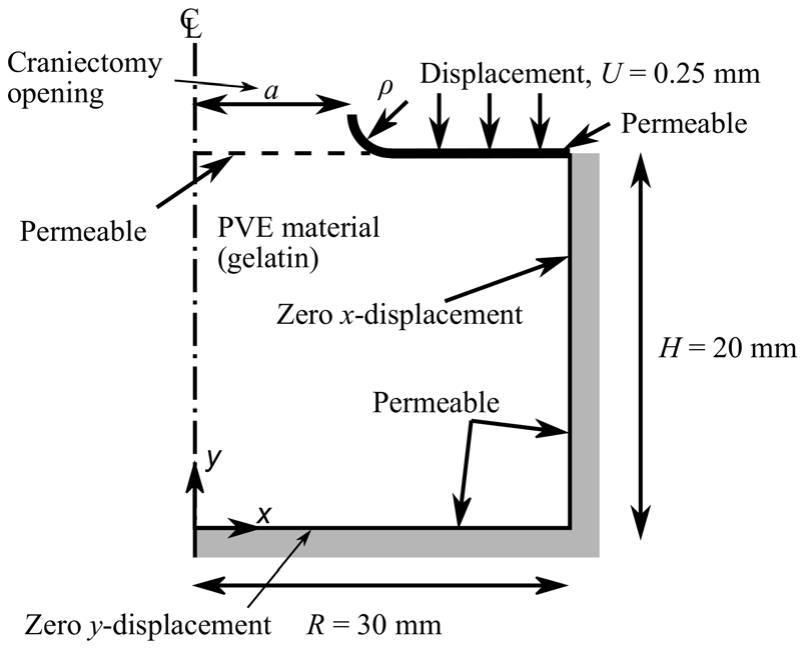
Schematic diagram of the idealised axisymmetric DC model geometry.

Figure 2 illustrates the axisymmetric FE model of an idealised craniectomy which was developed. The model has a similar cylindrical geometry and circular craniectomy opening as for the experimental model. The craniectomy opening 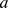 and the fillet radius 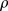 were defined as parameters to simplify a parametric study. The craniectomy opening 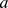 was varied between 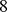 and 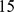 mm in 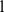 mm increments with 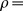 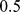 mm and the fillet radius 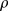 was varied from 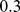 mm to 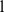 mm in 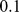 mm increments with 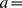 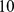 mm. The FE models with 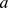 smaller than 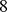 mm failed to converge due to excessive strains under the craniectomy edge. Abaqus Standard [21] with non-linear geometry was used in all simulations.

#### Materials

The measured material properties for gelatin given in Table 1 were used in a baseline set of FE calculations for comparison with the experiments. In addition, the influence of the poro-elastic and the visco-elastic components was analysed by varying material parameters from these base-line values. A linear PVE model was used as preliminary simulations showed that using a non-linear PVE model had little effect on the model response to loading, presumably as regions of high strains were comparatively small.

#### Mesh

Axi-symmetric quadratic elements with reduced integration (Abaqus element CAX
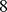
RPH) were used in all FE models. A fine mesh was created directly under the craniectomy edge with an average element length of 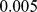 mm. This was graded to the edges where elements had a side length of 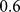 mm. The mesh used produced a force response within 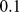
% of the converged mesh in a mesh refinement study, with the shear strain within 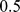
% of the converged value.

#### Loading and constraints

Displacement boundary conditions of zero 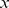-displacement on the vertical edges and zero 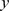-displacement on the bottom horizontal surface were applied, see Figure 2. The platen underwent a 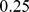 mm 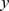 displacement at a speed of 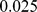 mm s^−1^ and was constrained to displace only in the vertical direction with no rotation. The platen was held at this position for 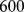 s. It was assumed that the interface between the PVE material and the platen was a frictionless, hard contact.

Boundaries were considered to be permeable in all simulations; this assumption is discussed in the results section.

## Results

### Finite element results

**Figure 3:**
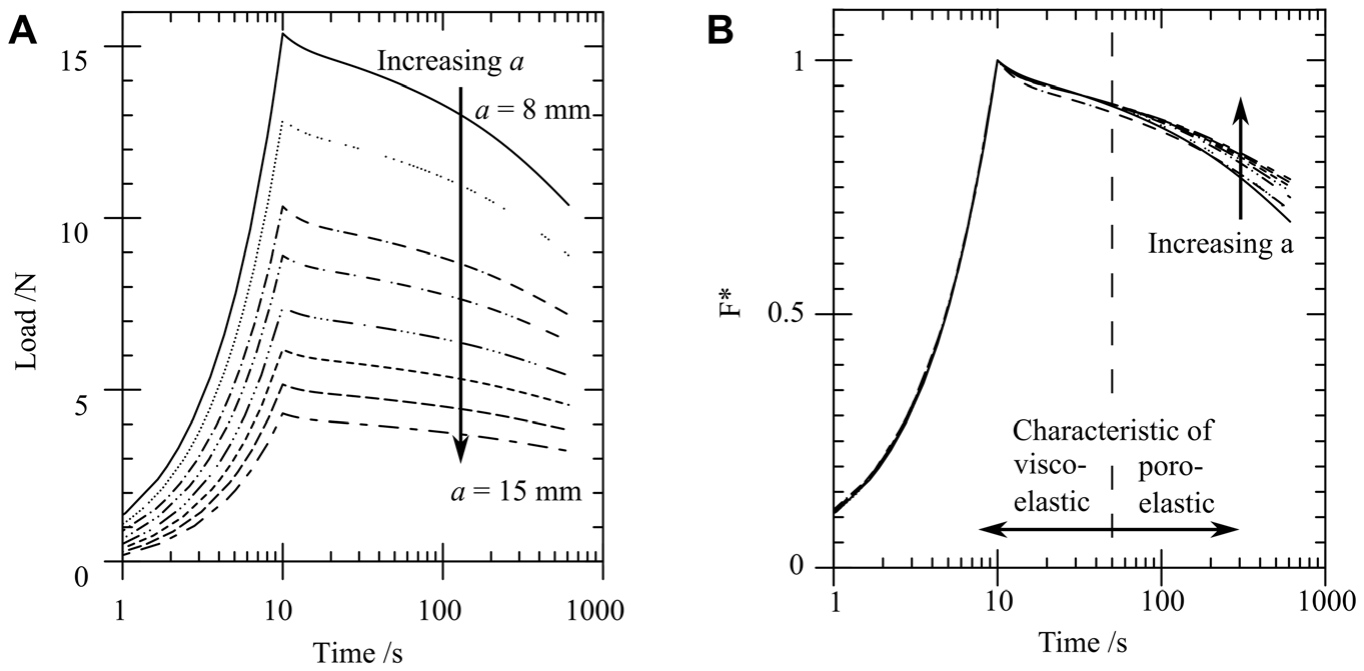
FE model load-time response for varying craniectomy opening, 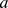. (A) actual load, (B) normalised load 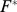.

Figure 3A shows the variation of load with time predicted by the FE model with varying values of craniectomy radius 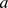 from 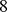 to 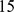 mm in steps of 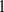 mm. There is a decrease in peak load with increasing craniectomy size. Associated with this there is a decrease in maximum shear strain 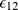 under the craniectomy from 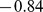 to 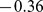 for 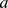 increasing from 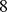 to 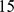 mm.

Load response curves were normalised by peak loads in order to analyse the effect of 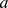, independent of the elastic response, as follows: 
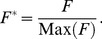
(7)


This normalisation, by eliminating the effect of craniectomy opening 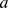 on peak load, highlights the shape of the response, see Figure 3B.

**Figure 4:**
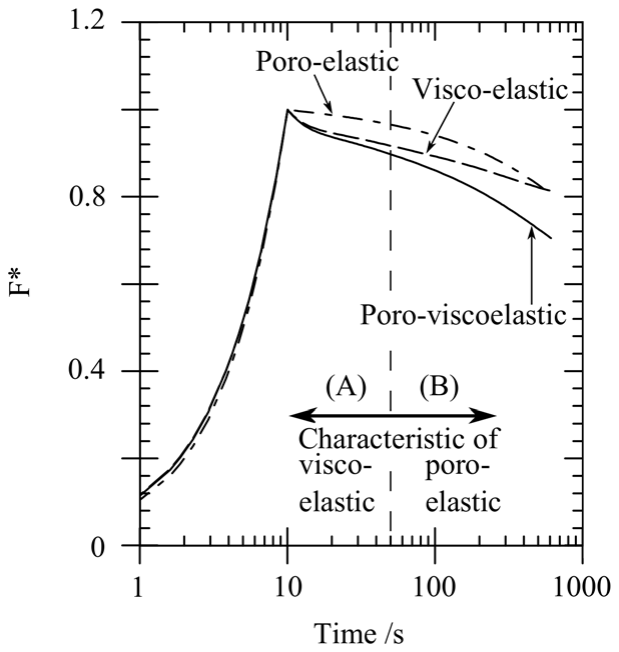
FE model results for normalised load versus time response. Normalised load versus time response of a visco-elastic, poro-elastic and poro-viscoelastic model with boundary conditions as per Figure 2. At early times (A) visco-elasticity dominates the response whilst at later times (B) poro-elasticity dominates.

Figure 4 compares the load-time response for visco-elastic, poro-elastic and poro-viscoelastic FE models having the same material properties as the gelatin PVE model. There are two distinct phases of relaxation. The first phase before a time of 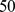 s has a shape characteristic of visco-elasticity, followed by a second poro-elastic consolidation phase after 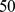 s. A similar form of load-time response was seen in Figure 3B for the effect of the craniectomy radius 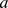. The curves of Figures 3B and 4 initially fall on a master curve but diverge with increasing time. The divergence occurs when the poro-elastic relaxation dominates the visco-elastic relaxation for 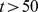 s. For these later times, an increase in craniectomy size 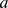 slows the relaxation of the sample.

**Figure 5:**
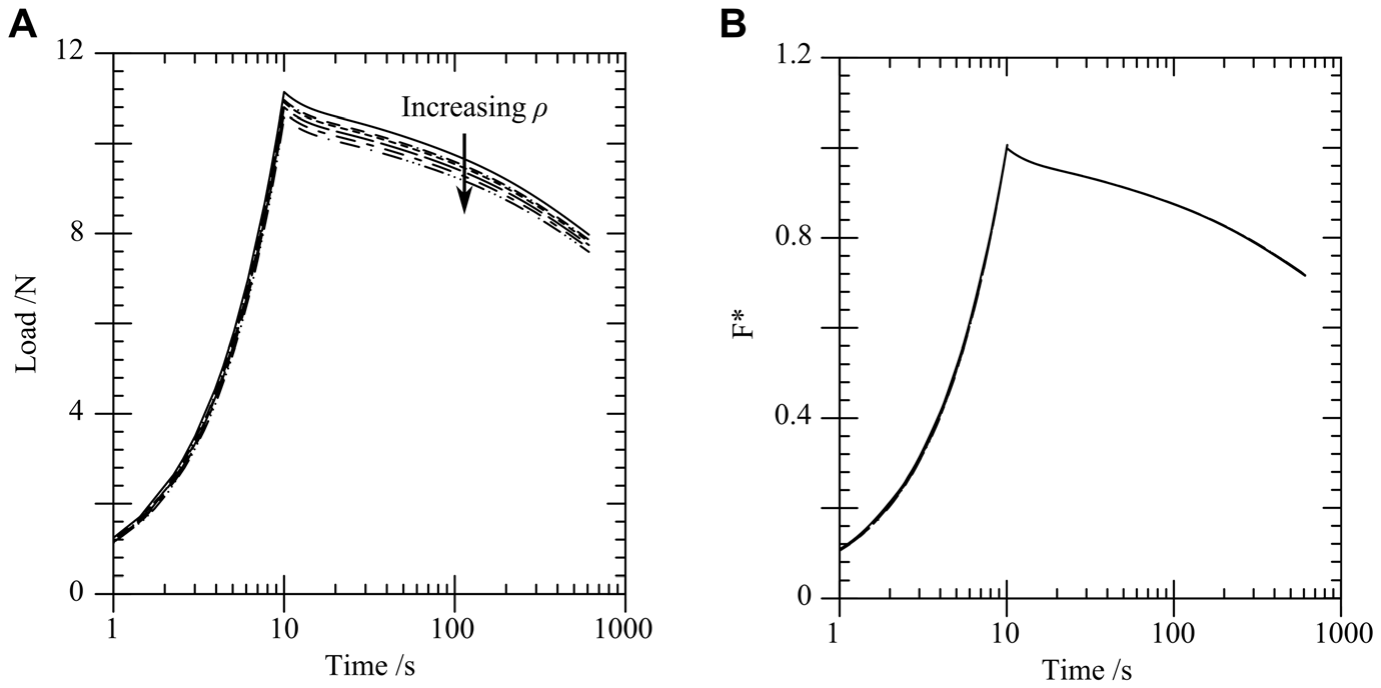
FE model load-time response for varying fillet radius, 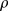. (A) actual load, (B) normalised load 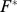.

Figure 5A shows that, as the fillet radius 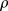 on the craniectomy increases, the force decreases slightly. The shapes of the curves in Figure 5A are similar and the normalisation of the force collapses the responses to a single curve, see Figure 5B. Hence changing 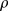 has little effect on the relaxation response of the material. However, the peak load and peak shear strain do depend on 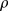. The peak strains 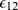 under the craniectomy decrease from 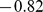 to 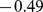 for 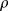 increasing from 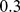 to 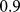 mm.

### Experimental results

**Figure 6:**
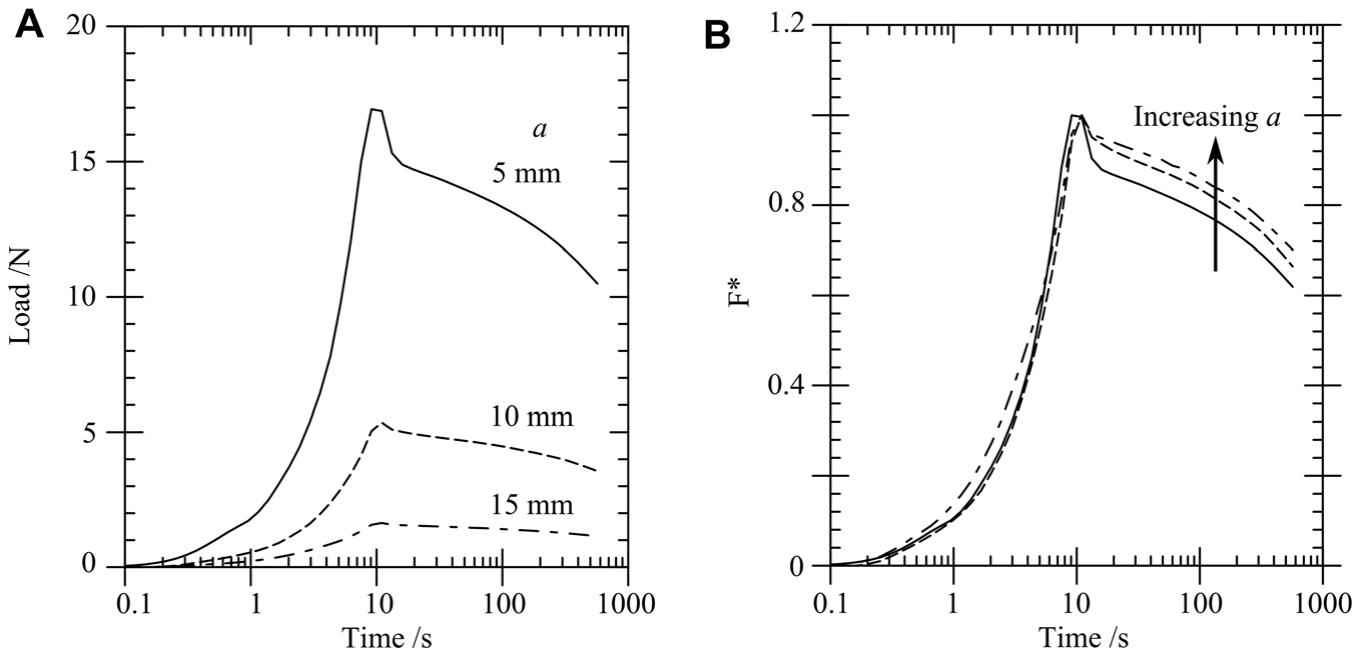
Experimental load versus time responses for varying craniectomy opening, 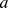. (A) Load versus time response, (B) experimental normalised load 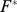 versus time response.

Figure 6A shows the load versus time response for individual tests at three values of 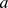 tested in the experimental model. There is a decrease in load as 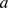 increases, with the same two-phase relaxation with the visco-elastic and poro-elastic portions.

Figure 6B shows the result of the normalisation of Equation 7 for the experimental results. The curves collapse in a similar manner to the FE results, with divergence from this single curve at later times.

### Comparison between FE and experimental models

#### Load comparison

**Figure 7:**
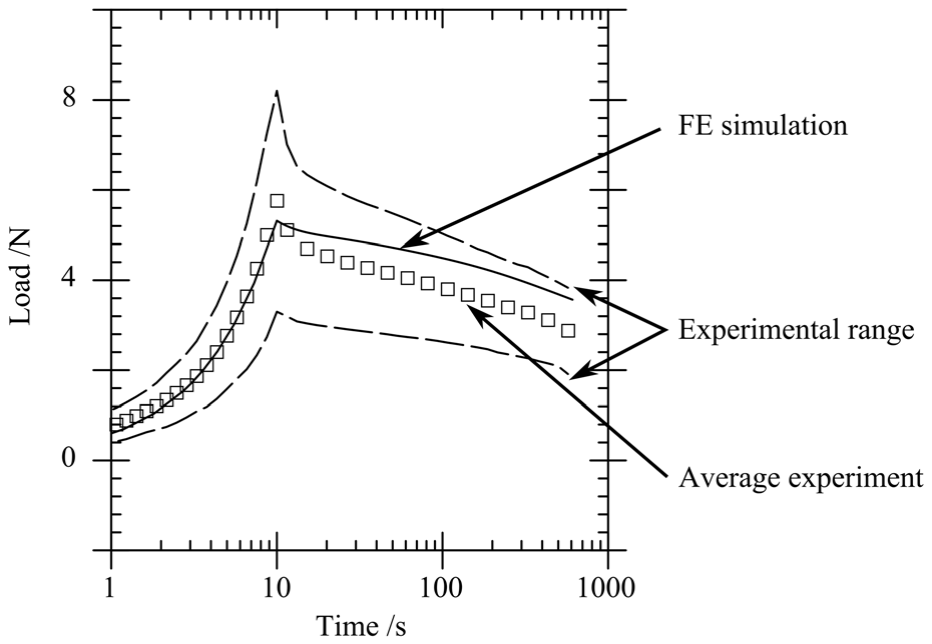
Load versus time response including average experimental response and FE simulation results for a craniectomy of 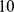 mm.

Figure 7 compares FE load response curves with the average experimental curves for 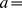 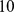 mm. The dashed lines in Figure 7 represent the average experimental results 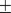 the standard deviation. The FE simulations follow a similar path to the experimental data, within the range of experimental results.

**Figure 8:**
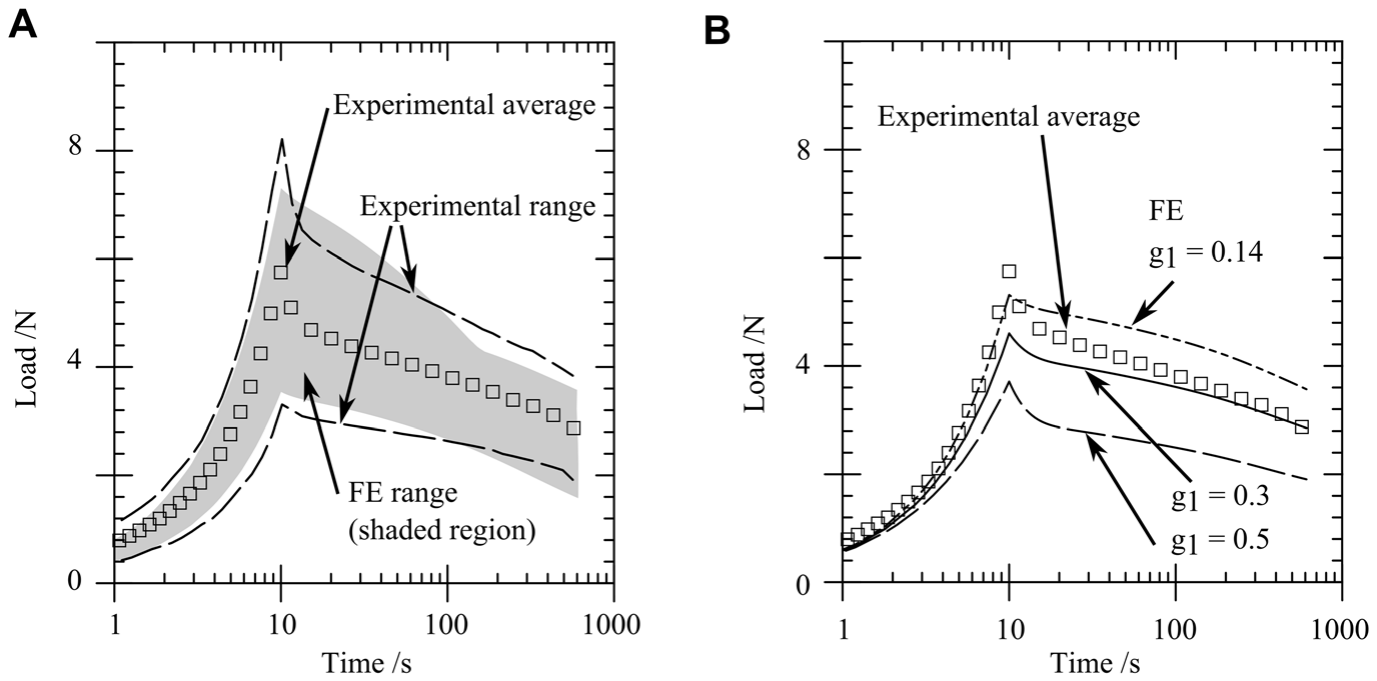
Sensitivity of the load versus time response to material parameters. (A) Load versus time response for a range of FE predictions using extreme values of the material parameters derived from unconfined and confined compression tests, (B) load versus time response showing a comparison of the experimental range with varying 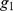 values.

Material parameters were varied in the FE model to match the variation in the parameters identified by compression tests, see Table 2. This variation in parameters produces a spread of FE curves which approximately reproduces that in the experiments (Figure 8A), suggesting that the experimental variation could be largely due to inherent variation in material parameters for gelatin.

**Table 2:** Extreme PVE parameters from unconfined and confined compression testing of gelatin, as used in Figure 8A.

	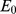 /kPa	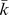 /mm s^−1^	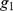	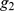	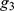	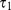 /s	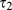 /s	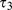 /s
Min.	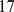	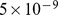	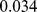	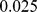	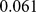	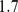	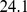	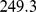
Max.	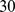	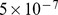	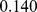	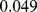	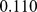	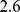	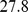	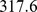

Figure 8B shows the effect of varying the dimensionless shear modulus 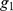 on the predicted load response. An increase in 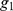 increases early relaxation (including during the loading phase) as seen in Figure 8B. The relaxation shape of the experiments can be matched by varying 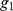. However, an increase in Young's modulus 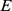 would be necessary in order to fully match the response if a larger 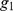 term were chosen.

#### Strain and deformation comparison

**Figure 9:**
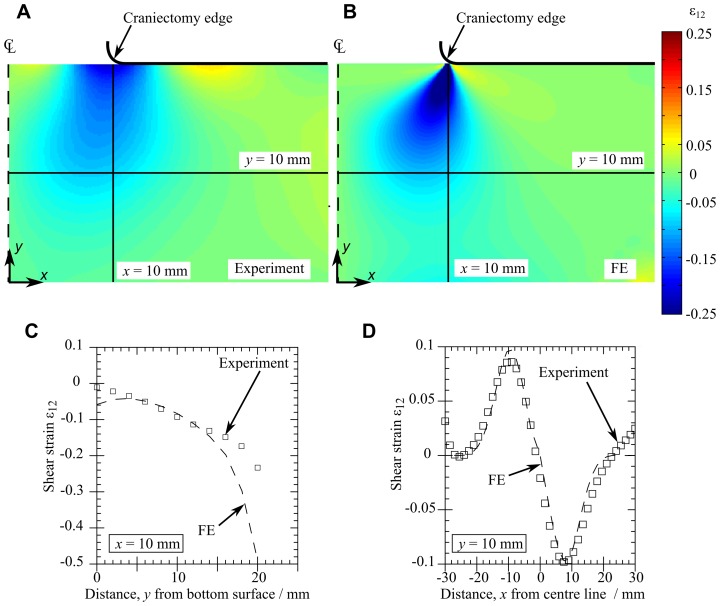
Shear strain 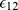 distribution for 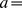 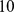 mm at time 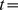 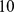 s. (A) Contour plot of the shear strain 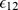 on the front face of the gelatin for a typical experiment, (B) contour plot of 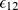 on the central plane of the FE model, (C) 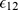 sampled along the line 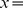 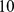 mm in Figure 9(A) and (B), (D) 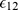 sampled along the line 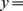 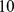 mm.

Figures 9A and B compare the experimental and FE shear strains on the front face (experiment) and central plane (FE) at the end of the loading step for a craniectomy radius 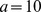 mm. Figures 9C and D plot the strain variation along the lines 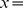 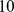 mm and 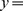 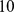 mm for both the experimental and FE cases.

The width, depth and overall shear strain values are comparable between the experimental and FE models. The FE model has smaller features of high shear strain in the region directly below the craniectomy edge (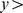 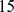 mm in Figure 9C). Results (not shown here) are similar for the larger 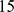 mm opening.

**Figure 10:**
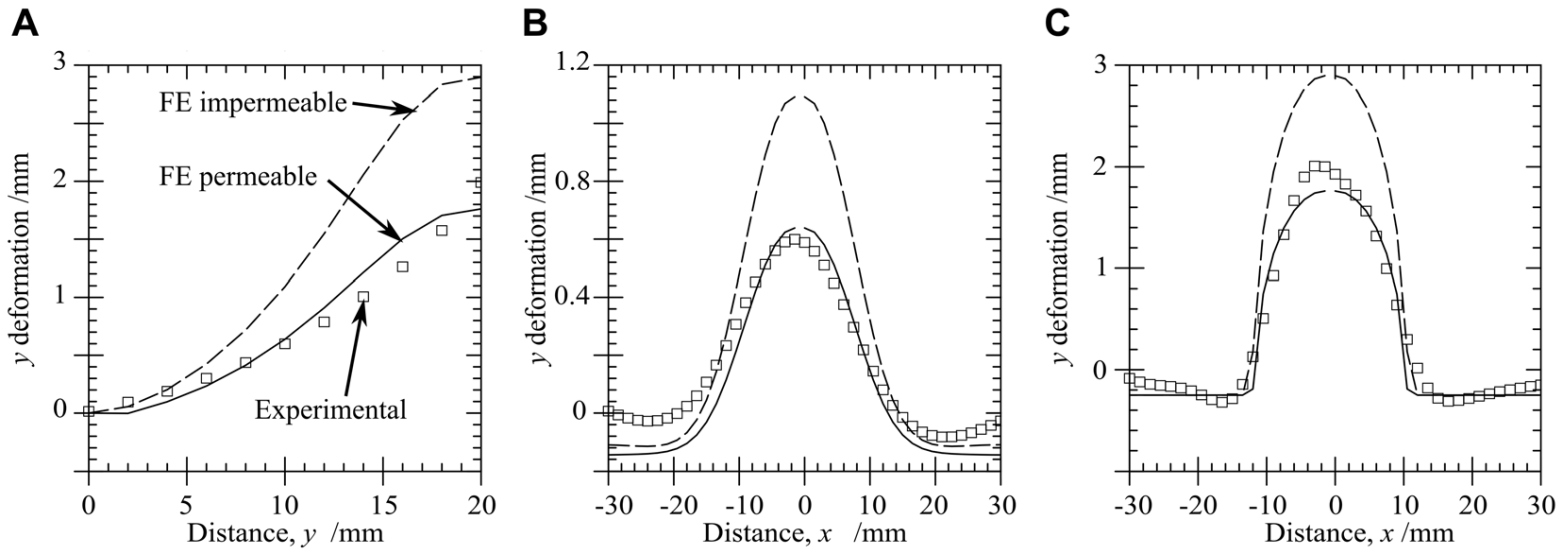
Comparison of experimental and FE deformation for 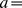 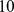 mm. Deformation in 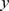 direction along: (A) 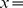 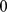 mm, (B) 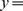 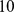 mm, (C) 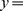 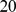 mm. Line types for (B) and (C) as per (A).

Figure 10A illustrates the vertical 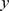-displacements sampled on a vertical centreline on the front face of the gelatin. Figures 10B and C show the 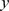-displacements along horizontal lines with 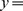 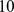 mm and 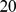 mm, i.e. the middle and the top surface of the front face. Along all sampled lines there is close agreement between the FE model with permeable boundaries and experiment.

#### Boundary conditions

The FE model with impermeable boundary conditions predicts deformations that are much larger in the bulge region, see Figure 10, and elevated loads (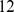 N as against 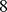 N for permeable condition and experiment). The results with a permeable condition are used in all other comparisons as this provided much closer agreement between the FE and experiments.

## Discussion

This paper develops an idealised FE model of DC, validating this with experimental measurements on a model with similar geometry. Whilst this study is a step towards understanding DC and the resultant strain, specific clinical inferences should not be taken from the model as the materials and dimensions of the model differ from the brain geometry. Rather the aim of the modelling work is to determine an appropriate modelling approach to be applied in the clinical application.

Complexities of using a PVE model are explored with constraints similar to DC. An area of particular interest is the mechanical strain applied to the brain under different conditions. This is especially important for clinicians, as the optimal parameters of craniectomy (particularly size and location) remain controversial.

Finite element predictions of the load-time response (Figure 7) match the experiment within experimental variability. The characteristic two-phase relaxation seen in experiments is also seen in the FE model. When only the peak load is required, Figure 4 shows that a visco-elastic model is adequate. From a clinical perspective the conditions at peak load are likely to correspond to the critical conditions leading to tissue damage associated with DC, which results indicate could be modelled using a visco-elastic approach. Only if modelling the strains during the subsequent transient event were important in predicting cell death would a PVE model be required to provide an accurate prediction (Figure 4). In this case it is likely that physiological factors would significantly modify the time-dependent material response, so that modifications to the PVE model would be required to capture these factors. Significantly smaller displacements have been applied in this model in comparison with those likely in clinical conditions. However the relative importance of viscoelastic and poro-viscoelastic deformations is expected to be similar at larger strains, so that the conclusions of this study can be applied to clinical models of the procedure.

Shear strains on the front face of the gelatin experimental model derived using image analysis were compared with FE predictions. There is a reasonable agreement between strains in the FE and experiment, although the spacing of the markers in the experimental tests precluded the measurement of the highest strain region as this region is smaller than the spacing between markers. It should be noted here that a linear PVE material has been used throughout the FE modelling, but peak shear strains in some simulations are higher than a linear limit. Comparison with a neo-Hookean model of the gelatin at the initial and infinite times (where 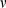 is known) revealed that, despite this limitation, the peak shear strains for the hyper-elastic case were within 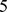
% of the equivalent linear elastic model (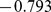 vs. 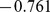). This highlights the need for preliminary FE models as presented in this paper to examine the modelling assumptions, showing in this case that a linear elastic model is adequate to capture the details of the strain response in DC. While the material properties of this study are not those appropriate to brain tissue, the strains observed in our tests are comparable with strains expected to cause microstructural damage in brain tissue (of the order of 0.05–0.35 [30]), confirming that the study explores a regime of deformation which is clinically relevant.

The modelling work assumed that the material was uniform and isotropic, matching the experimental behaviour of the gelatin. In practice brain mechanical properties will depend on position and are likely to exhibit an anisotropic dependence of damage on strain, associated with the anatomical and axonal structure of the brain. Wright and Ramesh [30] have shown in a study of traumatic brain injury how such factors can be incorporated into a model of brain damage associated with deformation in DC. A challenge in such work would be identifying the appropriate damage parameters associated with the timescales of deformation, although the micromechanical modelling approach of Cloots et al [31] provides an attractive way of helping determine these parameters.

It was shown that the permeable boundary condition matched the experimental data better than the impermeable condition. Flow on the boundary could be caused by a local dehydration of the gelatin. Although samples were kept in a sealed container, there may have been surface dehydration prior to testing. This surface dehydration could lead to a region of the gel which would allow the fluid from the interior to flow to the exterior in the same manner as a permeable boundary condition. Inspection of the boundary nodes in the FE models with permeable boundary conditions showed that, for a typical craniectomy size, the modelling predicts a fluid flow of 1.5 ml over the first 100 s of relaxation. This equates to a fluid build up of 0.15 mm over the surface of the model during that 100 s interval. In the experimental situation, the sample is kept hydrated and lubricated by applying a thin layer of water on the surface of the model prior to loading. It is therefore suggested that the small volume of fluid due to poro-elastic fluid flow could have been accommodated in the test rig despite the impermeable nature of the test rig walls. The permeable boundary may also replicate the surface of the brain. Fluid collections often develop on the surface of the brain near the craniectomy between the brain and the scalp [32] which may suggest a similar permeable surface behaviour. In any case results confirm that careful attention needs to be paid to this aspect of the model in the clinical application.

Parametric studies have highlighted the significant effects of craniectomy size and fillet radius on the deformation and force response for the assumed model material properties. Hence a study using brain-specific material properties should include these effects to provide guidance on optimising the clinical procedure for DC.

## Conclusions

The reasonable correlation between the FE model of the idealised craniectomy and the experimental model using gelatin confirms that the proposed FE model has the potential to provide useful predictions for the brain tissue strains developed during decompressive craniectomy procedures. The work has provided clear guidance on the requirements of such a model as applied to the clinical situation, in addition to the prerequisites of appropriate material and geometric properties. In order to obtain the peak response and critical strain during DC a visco-elastic approach is adequate; only to model the subsequent transient decay of strains is a poro-viscoelastic model required. In this case it is likely that physiological factors would significantly modify the time-dependent material response, so that modifications to the PVE model would be required to capture these factors. A linear elastic model is adequate to capture the peak strains during the procedure, but close attention should be paid to the boundary conditions at the brain/skull interface. In addition a clinical study should examine the effect of craniectomy opening and fillet radius on the developed strains.

## References

[1] Unterberg AW, Stover J, Kress B, Kiening KL (2004) Edema and brain trauma. Neuroscience 129: 1019–1027. 10.1016/j.neuroscience.2004.06.046 15561417

[2] Hutchinson PJ, Kolias AG, Czosnyka M, Kirkpatrick PJ, Pickard JD, et al (2013) Intracranial pressure monitoring in severe traumatic brain injury. BMJ: British Medical Journal 346: f1000. 23418278 10.1136/bmj.f1000

[3] Timofeev I, Santarius T, Kolias AG, Hutchinson PJ (2012) Decompressive craniectomy–operative technique and perioperative care. Advances and Technical Standards in Neurosurgery 38: 115–136. 22592414 10.1007/978-3-7091-0676-1_6

[4] Bor-Seng-Shu E, Figueiredo EG, Amorim RLO, Teixeira MJ, Valbuza JS, et al (2012) Decompressive craniectomy: a meta-analysis of inuences on intracranial pressure and cerebral perfusion pressure in the treatment of traumatic brain injury. Journal of Neurosurgery 117: 589–596. 22794321 10.3171/2012.6.JNS101400

[5] Timofeev I, Czosnyka M, Nortje J, Smielewski P, Kirkpatrick PJ, et al (2008) Effect of decompressive craniectomy on intracranial pressure and cerebrospinal compensation following traumatic brain injury. Journal of Neurosurgery 108: 66–73. 18173312 10.3171/JNS/2008/108/01/0066

[6] Hutchinson PJ, Corteen E, Czosnyka M, Mendelow AD, Menon DK, et al (2006) Decompressive craniectomy in traumatic brain injury: the randomized multicenter RESCUEicp study (www.RESCUEicp.com). Acta Neurochirurgica Supplement 96: 17–20. 16671415 10.1007/3-211-30714-1_4

[7] Cooper DJ, Rosenfeld JV, Murray L, Arabi YM (2011) Decompressive craniectomy in diffuse traumatic brain injury. New England Journal of Medicine 364: 1493–1502. 21434843 10.1056/NEJMoa1102077

[8] Cruz-Flores S, Berge E, Whittle IR (2012) Surgical decompression for cerebral oedema in acute ischaemic stroke. doi:10.1002/14651858.cd003435.pub2. Cochrane Database of Systematic Reviews. 10.1002/14651858.CD003435 12137695

[9] Kolias AG, Kirkpatrick PJ, Hutchinson PJ (2013) Decompressive craniectomy: past, present and future. Nature Reviews Neurology 9(7): 405–15. 23752906 10.1038/nrneurol.2013.106

[10] Elkin BS, Morrison B (2007) Region-specific tolerance criteria for the living brain. Stapp Car Crash Journal 51: 127–38. 18278594 10.4271/2007-22-0005

[11] Miller K, Wittek A, Joldes G, Horton A, Dutta-Roy T, et al (2010) Modelling brain deformations for computer-integrated neurosurgery. International Journal for Numerical Methods in Biomedical Engineering 26: 117–138.

[12] Gao CP, Ang BT (2008) Biomechanical modeling of decompressive craniectomy in traumatic brain injury. Acta Neurochirurgica Supplement 102: 279–282. 10.1007/978-3-211-85578-2_52 19388329

[13] Cheng S, Bilston LE (2007) Unconfined compression of white matter. Journal of Biomechanics 40: 117–24. 16376349 10.1016/j.jbiomech.2005.11.004

[14] von Holst H, Li X, Kleiven S (2012) Increased strain levels and water content in brain tissue after decompressive craniotomy. Acta Neurochirurgica: 1–11. 10.1007/s00701-012-1393-2 22648479

[15] Kyriacou SK, Miller K, Mohamed A, Neff S (2002) Brain mechanics for neurosurgery: Modeling issues. Biomechanics and Modeling in Mechanobiology 1: 151–164. 14595547 10.1007/s10237-002-0013-0

[16] Franceschini G (2006) The Mechanics of Human Brain Tissue. PhD. thesis, University of Trento.

[17] Kalyanam S, Yapp RD, Insana MF (2009) Poro-viscoelastic behavior of gelatin hydrogels under compression – implications for bioelasticity imaging. Journal of Biomechanical Engineering 131: 081005. 19604017 10.1115/1.3127250

[18] Ivarsson J, Viano DC, Lövsund P, Aldman B (2000) Strain relief from the cerebral ventricles during head impact: Experimental studies on natural protection of the brain. Journal of Biomechanics 33: 181–9. 10653031 10.1016/s0021-9290(99)00144-x

[19] Zhang J, Yoganandan N, Pintar FA, Guan Y, Gennarelli TA (2007) Experimental model for civilian ballistic brain injury biomechanics quantification. Journal of Biomechanics 40: 2341–2346. 17166502 10.1016/j.jbiomech.2006.10.021

[20] Galli M, Oyen ML (2008) Spherical indentation of a finite poroelastic coating. Applied Physics Letters 93: 031911.

[21] Dassault Systèmes Simulia (2011) Abaqus User Manual 6.11.1–Section 21.7.1. Vélizy-Villacoublay, France.

[22] Suh JK, DiSilvestro MR (1999) Biphasic poroviscoelastic behavior of hydrated biological soft tissue. Journal of Applied Mechanics 66: 528–535.

[23] Lakes R (2009) Viscoelastic Materials. Cambridge University Press. doi: 10.1017/CBO9780511626722.

[24] Strange DGT, Oyen ML (2011) Composite hydrogels for nucleus pulposus tissue engineering. Journal of the Mechanical Behavior of Biomedical Materials 11: 16–26. 22658151 10.1016/j.jmbbm.2011.10.003

[25] Detournay E, Cheng A (1993) Fundamentals of poroelasticity, in Comprehensive rock engineering: Principles, practice and projects, Vol. II, Analysis and design method, Fairhurst, C., ed, New York, Pergamon: 113–171.

[26] Miller K, Chinzei K (1997) Constitutive modelling of brain tissue: experiment and theory. Journal of Biomechanics 30: 1115–1121. 9456379 10.1016/s0021-9290(97)00092-4

[27] Crocker J (1996) Methods of digital video microscopy for colloidal studies. Journal of Colloid and Interface Science 179: 298–310.

[28] Hert S, Seel M (2010) dD convex hulls and delaunay triangulations. In: CGAL User and Reference Manual, CGAL Editorial Board. 3.6 edition.

[29] Screen HRC, Evans SL (2009) Measuring strain distributions in the tendon using confocal microscopy and finite elements. The Journal of Strain Analysis for Engineering Design 44: 327–335.

[30] Wright RM, Ramesh KT (2011) An axial strain injury criterion for traumatic brain injury. Biomechanics and Modelling in Mechanobiology 11: 245–260. 10.1007/s10237-011-0307-1 21476072

[31] Cloots RJH, van Dommelen JAW, Nyberg T, Kleiven S, Geers MGD (2011) Micromechanics of diffuse axonal injury: inuence of axonal orientation and anisotropy. Biomechanics and Modelling in Mechanobiology 10: 413–422. 10.1007/s10237-010-0243-5 20635116

[32] Nalbach SV, Ropper AE, Dunn IF, Gormley WB (2012) Craniectomy-associated progressive extraaxial collections with treated hydrocephalus (capecth): Redefining a common complication of decompressive craniectomy. Journal of Clinical Neuroscience 19: 1222–1227. 22727206 10.1016/j.jocn.2012.01.016

